# Assessment of age, gender, and anxiety on ECG waveform morphology in a large population of domestic dogs

**DOI:** 10.1038/s41598-022-11378-3

**Published:** 2022-05-05

**Authors:** Lisa Murphy, Reid Nakamura, Jessica Gentile-Solomon, Allison Spake, Donald Szlosek

**Affiliations:** 1 Friendship Hospital for Animals, 4105 Brandywine St NW, Washington, DC 20016 USA; 2 IDEXX Laboratories, Inc., Westbrook, ME USA

**Keywords:** Cardiology, Medical research

## Abstract

Cardiovascular diseases are major causes of death in the western world and this incidence increases in the elderly population. With aging, there are physiologic changes to the cardiac structure secondary to adipose tissue deposition, calcification of valve leaflets and changes in the structure of the heart including atrial remodeling. Such changes can make the myocardium more susceptible to stress leading to a higher prevalence of cardiovascular diseases in the aging population. Studies in healthy humans have shown that these structural and molecular changes in the heart are manifested as changes on an electrocardiogram (ECG). Using animal models, similar ECG changes have been found in guinea pigs, rabbits, and mice. No veterinary study has specifically evaluated if comparable aging changes occur in canine species. In this cross-sectional retrospective study, 12,026 ECGs from apparently healthy dogs were obtained and evaluated. Age was observed to have both linear and non-linear associations with multiple ECG variables, including P wave amplitude and duration, R amplitude and QRS duration. This study confirmed that, like humans, there may be ECG changes secondary to normal physiological cardiac aging. Further studies are warranted to confirm and elaborate on these findings as canines may be a useful model for cardiac aging in humans.

## Introduction

The frequency of cardiovascular disease increases in aging dogs and is one of the most common causes of death in older dogs^1^. However this must be differentiated from cardiac aging which is characterized by gradual alterations in myocardial structure and function without structural cardiac disease^2^. Studies of cardiac aging in people are difficult due to various confounding factors ranging from genetic, ethnic, lifestyle and environmental factors which may modulate cardiac aging, however true aging effects can be seen at both macroscopic and microscopic levels^3,4^. Such changes in humans include epicardial adipose tissue deposition, calcification of valve leaflets and changes in the structure of the heart including atrial remodelling^5,6^. There are also abnormalities in ventricular compliance in people as they age as a result of collagen deposition and diffuse interstitial fibrosis which can lead to depressed systolic and diastolic function^6–9^. This is reflected in the electrocardiogram (ECG), and alterations in P wave and QRS morphology are identified in normal people as they age^10–13^.

Animal models have been used to evaluate cardiac aging, especially in murine species where rats and mice at 24 months old are comparable to an 85 year old human^14^. Ventricular hypertrophy is commonly found in aging people, male rodents, guinea pigs and rabbits; however a study of a beagle dog colony aged 3 to 14 years of age failed to identify similar changes^3,15,16^. Slowed conduction and prolongation of the QRS complexes are noted in aging humans and animals due to reduced cell-to-cell connections following reduced expression of gap junction proteins like connexion-43^17,18^. Prolonged action potential duration was noted in older canine cardiomyocytes contributing to slower electrical recovery of the myocardium^3^. Few veterinary studies have evaluated age related changes on the ECG, although one previous study showed that both QRS duration and R wave amplitude decreased with age when comparing young and geriatric German Shepherds^1^. This R wave change was also noted in a study evaluating aging in military working dogs^19^. Other smaller veterinary studies did find any significant correlation between age and ECG changes^20,21^. Given this data and previous studies in humans, it appears likely that dogs without structural cardiac disease experience changes to their ECG morphology as they age as well. Therefore, the purpose of this study was to determine if P wave and QRS morphology changed in apparently healthy dogs as they aged and to assess if aspects such as anxiety level, heart rate, sex, mean electrical axis (MEA), or body weight also affected these ECG morphologies.

## Statistical analysis

Continuous variables were reported as median and IQR and categorical variables were reported as percentage and frequency. Multivariable-adjusted linear regression analyses were used to model a flexible association between each of the five ECG waveform measurements (P amplitude, R amplitude, P-wave duration, R-wave duration, and QRS-wave duration). Continuous covariates consisted of age, weight, MEA, and heart rate (all modeled using restricted cubic splines). Categorical covariates consisted of reported anxiety level (ref: “Not anxious/very calm”, “Average Anxiety”, “Very anxious or nervous”) and sex (ref: “female”, ”female/spayed”, ”male”, ”male/neutered”). Knot selection for restricted cubic splines was selected by Akaike Information Criteria (AIC). Model performance was assessed using bootstrap calibration curves and AIC (Supplemental Figure S2)^22,23^. Anxiety was decided *pre-hoc* to test for an interaction between the other covariates and the ECG waveforms. A *post-hoc* exploratory analysis of the five ECG waveform models was conducted with the addition of breed as a covariate (see Supplemental Methods and Supplemental Figures S4–S11). Univariate linear associations were tested using Spearman’s rank correlation test (Spearman’s $\rho$). All data analyses were conducted using R version 4.0.4. Univariate linear associations were tested using Spearman’s rank correlation test (Spearman’s ). All data analyses were conducted using R version 4.0.4^24^. Data analysis was done with the tidyverse and multiple helper functions within the following packages: data.table, magrittr, Hmisc^2–5,25–28^. Calibration curves and regression modeling were performed using the rms package^6,29^. Statistical significance was set at P < 0.05. Data analysis was done with the tidyverse and multiple helper functions within the following packages: data.table, magrittr, Hmisc. Calibration curves and regression modeling were performed using the rms package. Statistical significance was set at P < 0.05.

## Results

A total of 50,000 ECGs were screened, of which 75.9% (37,974/50,000) were excluded from the study leaving 12,026 ECGs as the total study population. The median age of the study population was 7.8 years (IQR: 2.8–10.6) and the median weight was 12.5 kg (6.3–27.0 kg). The top dog breeds were: Mixed Breeds (n = 2,941), Chihuahua (n = 707), Labrador Retriever (n = 675), Yorkshire Terrier (n = 540), and Shih Tzu (497). Additional demographic information can be found in Table 1, Supplemental Table S2, and Supplemental Figure S3. A weak negative linear relationship was observed between age (year) and weight (lbs, Spearman’s $\rho$ = − 0.081, P < 0.001, Supplemental Figure S3). As an exploratory analysis, assessment of ECG waveforms and covariates for the top breeds are shown (Supplemental Figures S4–S11).

**Table 1:** Categorical Demographic Information.

Category	%	N
**Sex**		
Female	21.2	2548
Female, Spayed	29.8	3588
Male	21.4	2572
Male, Neutered	27.6	3318
**Anxiety**		
Not anxious/very calm	7.8	937
Average Anxiety	62.6	7525
Very anxious or nervous	29.6	3564

### P-Wave Amplitude

**Figure 1:**
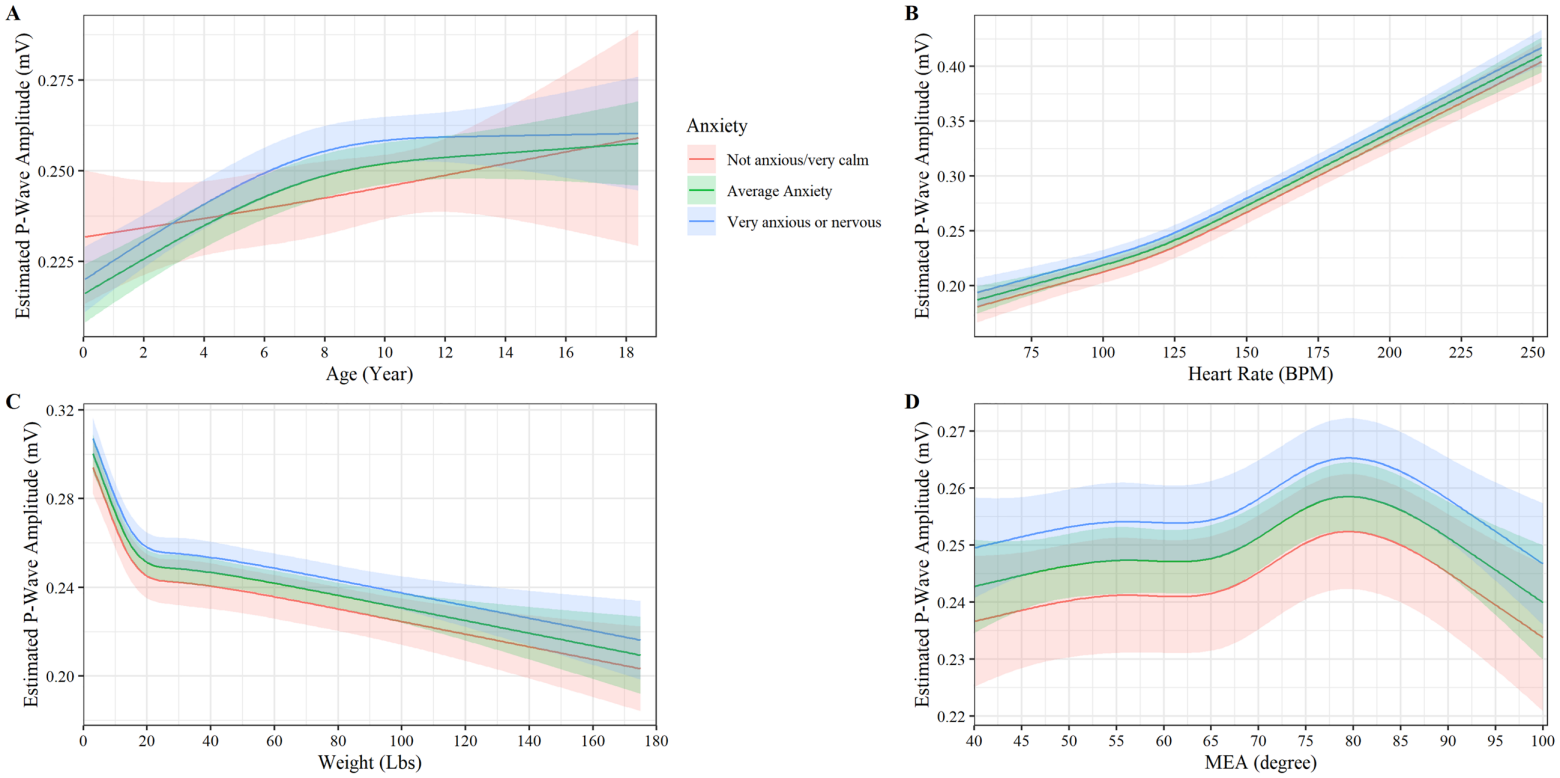
Plot of the p-wave amplitude (mV) versus (**A**) age, (**B**) heart rate, (**C**) weight, and (**D**) MEA by anxiety. The y axis shows the estimated p-wave amplitude from the fully adjusted model for observed values while holding the values of covariates at their referent values. The curves display the trend in p-wave amplitude with increase age, heart rate, weight, and MEA. Shaded regions represent the 95% confidence interval of the prediction estimates from the regression model.

Age was observed to have a non-linear relationship with P-wave amplitude for anxious and very anxious dogs with estimated P-wave amplitude increasing steadily until approximately eight years of age, where the curve levels off (P < 0.001, Fig. 1A). While an age-anxiety interaction was found with non-anxious dogs seeing a more linear relationship with age compared to anxious/very anxious dogs, the effect was small with very anxious dogs observed to have a higher P-wave amplitude than non-anxious dogs ages 4.5 to 12 years old (P-Value < 0.048, Fig. 1A). Heart rate had a near linear relationship with P-wave amplitude; a small increase in slope was noted at approximately 130 beats per minute (P < 0.001, Fig. 1B). No interaction between heart rate and anxiety levels on P-wave amplitude was found (P = 0.690, Fig. 1B). Weight had a strong negative non-linear relationship with P-wave amplitude (P < 0.001, Fig. 1C). P-wave amplitude decreased steeply up to 9 kg and then decreased slower as weight increased. No interaction between weight and anxiety levels was observed to affect P-wave amplitude (P = 0.075, Fig. 1C). MEA axis was observed to have a non-linear relationship with P-wave amplitude. P-wave amplitude steadily increases until an MEA of 55 degrees and then increases more rapidly until it peaks at an MEA of 78 degrees and starts to decline (P < 0.018, Fig. 1D). No interaction between MEA and anxiety levels was observed to affect P-wave amplitude (P = 0.792, Fig. 1D). Neuter status was observed to have an impact on P-wave amplitude with neutered dogs having higher P-wave amplitude than non-neutered animals (P < 0.001).

### R-wave amplitude

**Figure 2:**
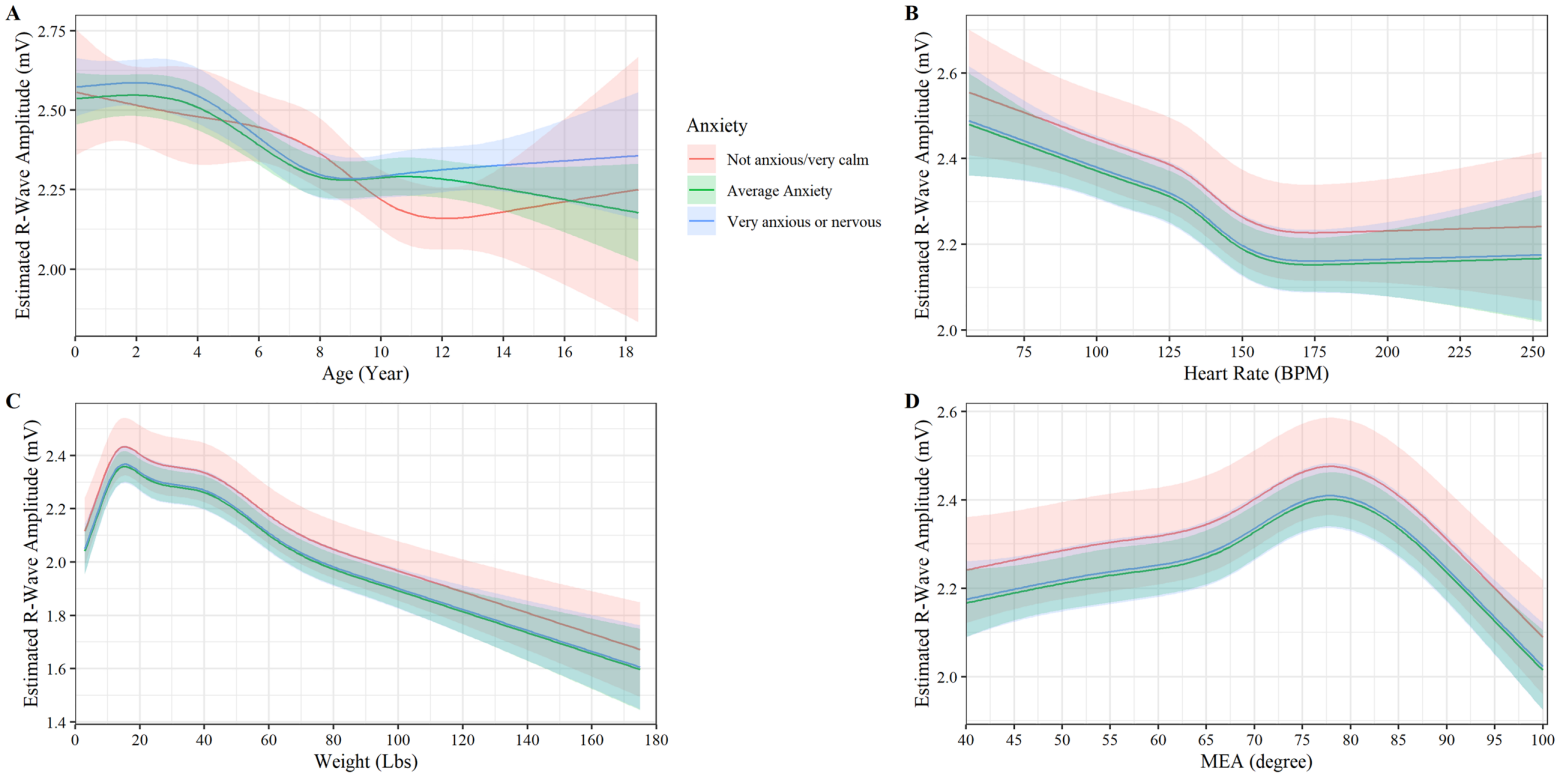
Plot of the r-wave amplitude (mV) versus (**A**) age, (**B**) heart rate, (**C**) weight, and (**D**) MEA by anxiety. The y axis shows the estimated r-wave amplitude from the fully adjusted model for observed values while holding the values of covariates at their referent values. The curves display the trend in r-wave amplitude with increase age, heart rate, weight, and MEA. Shaded regions represent the 95% confidence interval of the prediction estimates from the regression model.

R-wave amplitude had a non-linear relationship with age (P < 0.001, Fig. 2A). Among very anxious and average-anxious dogs, R-wave amplitude was stable at approximately 2.5 mV from birth to 3.5 years of age and then steadily declines from 3.5 years to 8 years of age until stabilizing at 2.25 mV (P < 0.001, Fig. 2A). For very anxious dogs, this decline continues until 10.5 years old and steadily increases out to 18 years of age, although a lot of variation is noted, and the age-anxiety interaction effect is minimal (P < 0.047). Heart rate had a weak negative non-linear relationship with R-wave amplitude with a small “dip” in slope at 130 beats per minutes that levels off at approximately 2.15 mV and 150 beats per a minute (P < 0.046, Fig. 2B). No interaction between heart rate and anxiety levels was observed to affect R-wave amplitude (P = 0.219, Fig. 1B). R-wave amplitude increases linearly with bodyweight until approximately 8 kg, then steadily decreases (P < 0.001, Fig. 2C). No interaction between weight and anxiety levels was observed to affect R-wave amplitude (P = 0.940, Fig. 2C). MEA had a non-linear relationship with R-wave amplitude, peaking at approximately 78 degrees and then decreasing linearly as MEA increased (P < 0.002, Fig. 2D). No interaction between MEA and anxiety levels was observed to affect R-wave amplitude (P = 0.379, Fig. 2D). Sex and neuter status were observed to have a small impact on R-wave amplitude with neutered dogs having higher R-wave amplitude than non-neutered animals (P < 0.001) and male dogs having higher R-wave amplitude than female dogs (P < 0.001).

### P-wave duration

**Figure 3:**
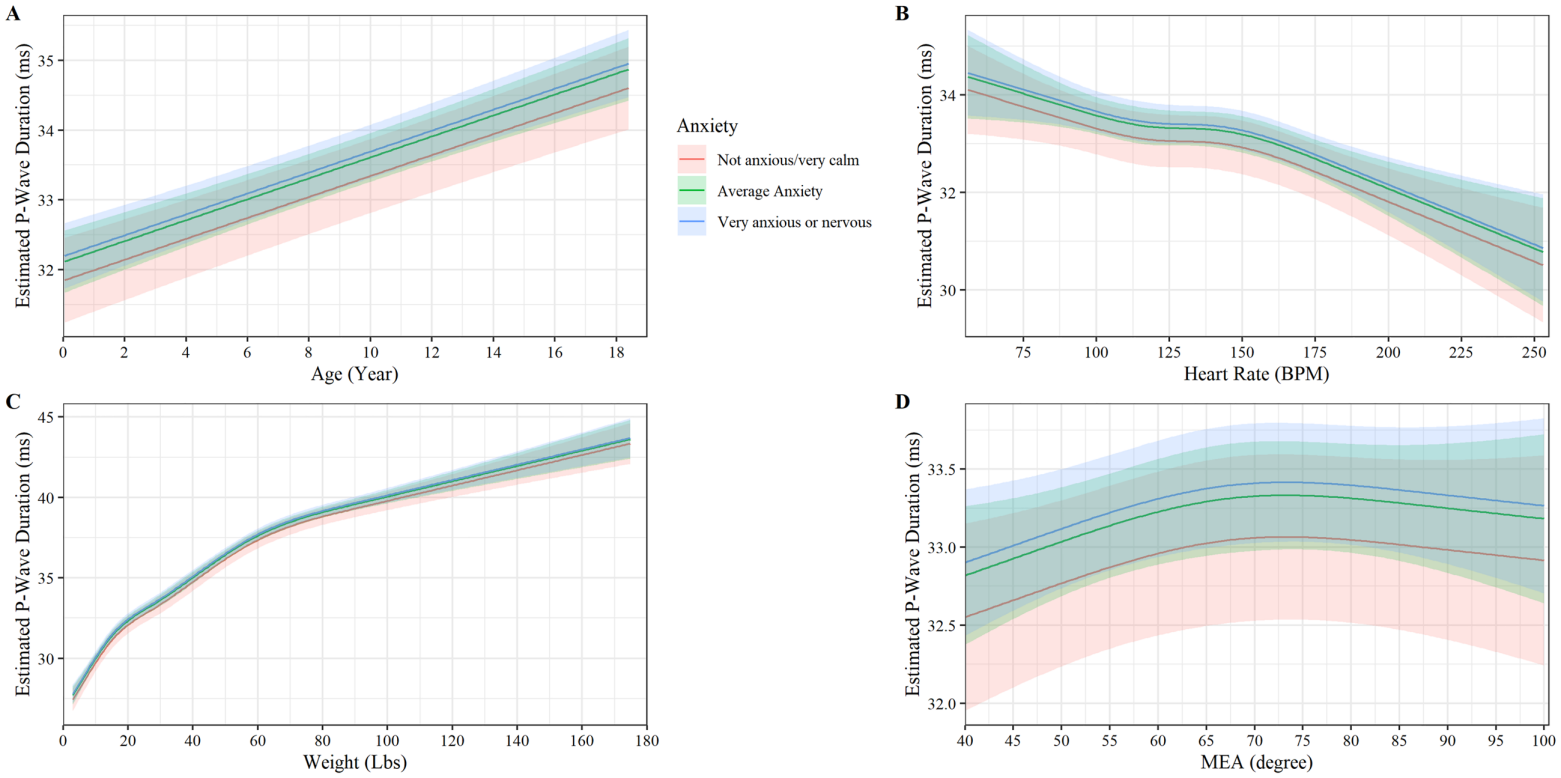
Plot of the p-wave duration (ms) versus (**A**) age, (**B**) heart rate, (**C**) weight, and (**D**) MEA by anxiety. The y axis shows the estimated p-wave duration from the fully adjusted model for observed values while holding the values of covariates at their referent values. The curves display the trend in p-wave duration with increase age, heart rate, weight, and MEA. Shaded regions represent the 95% confidence interval of the prediction estimates from the regression model.

P-wave duration had a strong positive linear relationship with age (slope 0.15 P wave duration (ms)/ year of age, P < 0.001, Fig. 3A). No age-anxiety interaction was observed to affect P-wave duration (P = 0.764). A non-linear association between P-wave duration and heart rate was observed with P-wave duration decreasing linearly with increasing heartrate until 130 beats per minute where P-wave duration plateaus at 33.5 ms until decreasing linearly again at approximately 150 beats per minute (P < 0.030, Fig. 3B). No heart rate-anxiety interaction affected P-wave duration (P = 0.272, Fig. 3B). Weight was observed to increase non-linearly in a curvilinear relationship with P-wave duration until approximately 27 kg and 37.5 ms where is starts to increase linearly (P < 0.001, Fig. 3C). No weight-anxiety interaction was affected P-wave duration (P = 0.836, Fig. 3C). MEA had a weak non-linear association with P-wave duration, increasing linearly until approximately an MEA of 65 degrees then leveling off (P < 0.028, Fig. 3D). No MEA-anxiety interaction with P-wave duration was observed (P = 0.547). Sex was observed to have a small impact on P-wave duration with male dogs having lower P-wave duration than female dogs (P < 0.001). Neuter status did not have an impact on P-wave duration (P < 0.963).

### QRS-wave duration

**Figure 4:**
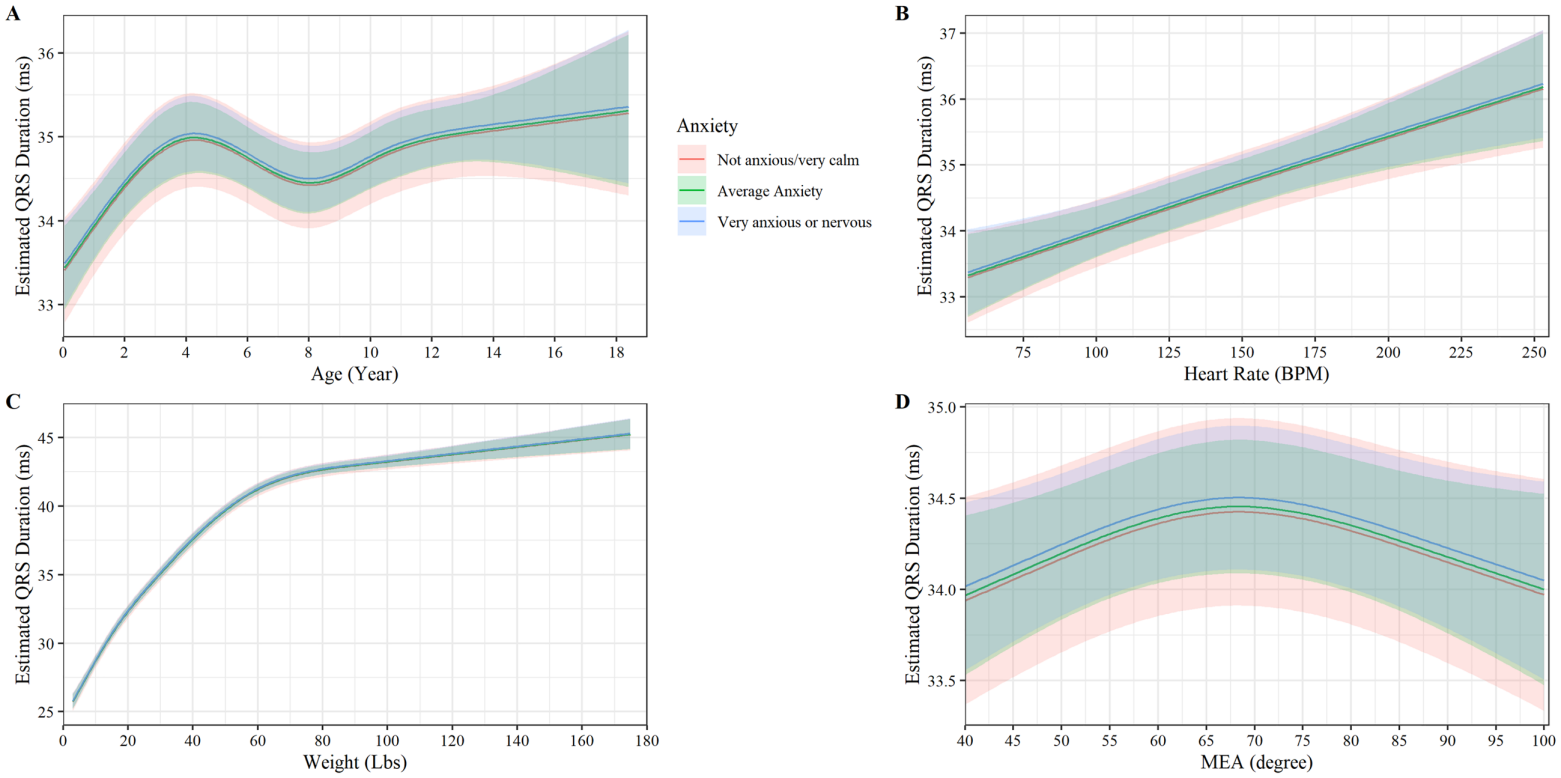
Plot of the QRS-wave duration (ms) versus (**A**) age, (**B**) heart rate, (**C**) weight, (**D**) MEA by anxiety. The y axis shows the estimated QRS -wave duration from the fully adjusted model for observed values while holding the values of covariates at their referent values. The curves display the trend in QRS-wave duration with increase age, heart rate, weight, and MEA. Shaded regions represent the 95% confidence interval of the prediction estimates from the regression model.

QRS-wave duration had a non-linear relationship with age, increasing linearly until about 4 years old, then decreasing slightly until increasing linearly once again around 8 years old (P < 0.001, Fig. 4A). No age-anxiety interaction was observed to affect QRS-wave duration (P = 0.770). A positive linear relationship between QRS-wave duration and heartrate was found (slope = 0.15 ms QRS-wave duration/10 beats per minute, P < 0.001 Fig. 4B). No heartrate-anxiety interaction impacted QRS-wave duration (P = 0.367, Fig. 4B). Weight increased non-linearly in a curvilinear relationship with QRS-wave duration, until 36 kg and 38 ms when it starts to increase linearly (P < 0.001, Fig. 4C). No weight-anxiety interaction was observed to affect QRS-wave duration (P = 0.929, Fig. 4C). MEA had a non-linear relationship with QRS-wave duration following a parabolic curve with a peak at 68 degrees (P < 0.009, Fig. 4D). No MEA-anxiety interaction was observed to affect QRS-wave duration (P = 0.972, Fig. 4D). Sex and neuter status did not impact QRS-wave duration (P = 0.107).

## Discussion

Similar to humans, this study identified several ECG changes in apparently healthy dogs associated with increasing age. Both P wave duration and P wave amplitude were observed to have a positive association with age. Prolonged P wave duration has been noted commonly in geriatric humans^10,30,31^. P wave duration correlates well to intra-atrial conduction time and mainly depends on conduction velocity and atrial size^32^. The amplitude of the P wave is primarily affected by initial right atrial depolarization while the duration is caused by activation of the left atrium^33^. Clinical differentials for pathologic increases in P wave duration include left atrial enlargement although this is a relatively insensitive finding as many dogs with markedly enlarged left atriums on echocardiographic exam can have a normal P wave duration on ECG^34^. An increased P wave duration could be indicative of age-associated atrial enlargement or some degree of reduced conduction^35^. The clinical implications of such findings are not known in dogs; however, in humans this is considered to lead to an increased susceptibility to atrial fibrillation^35,36^.

One previous veterinary study compared the P wave dispersion (PWD) between healthy dogs and those with chronic valvular disease (CVD)^16^. PWD is the difference between the maximum and minimum P wave duration recorded from different ECG leads. Both the duration of the P wave and the PWD are thought to reflect the electrophysiologic properties of the atrial muscle. No direct relationship between a higher PWD was noted in older dogs although a tendency for a greater spread of PWD was seen more commonly in healthy dogs over 8 years of age. The study also found increased PWD was significantly more common in dogs with CVD than healthy dogs. As noted in older humans, the authors postulated that increased amounts of fibrosis in the area could lead to abnormalities in the propagation of impulses in this area which could increase the PWD^10,35,37^. PWD was not evaluated in this study although a study looking at this indicator in a larger cohort of dogs in different age groups is warranted.

Electrocardiographic changes in the P wave likely reflects atrial remodeling. This process is common in humans over eighty years of age in those without structural cardiac disease and may promote the occurrence of arrhythmias including atrial fibrillation^38,39^. There are conflicting studies in humans as to the effect of aging on the atrial excitability refractory period (ERP) with some studies showing the ERP is increased and others have found the ERP remains unchanged^38–40^. In a study of older canine atria, the conduction velocity was reduced with a concurrent slowing in response to premature stimuli but not to sinus beats^36^. In humans over the age of eighty, some studies have shown increases in left atrial (LA) volume in people without concurrent cardiac disease^41^. However, some cardiac changes secondary to aging are noted earlier in life in individuals over sixty. In people over sixty, diastolic changes from altered left ventricle (LV) filling have been demonstrated^6,14,31,42^. Normal aging leads to myocardial fibrosis, altered collagen and abnormal calcium handling within the myocytes which is postulated to lead to reduced left ventricular compliance^9,31,41,43^. Given that LA enlargement develops later, it is not known if the LV compliance lead to changes in LA volume^44^. Altered LV relaxation can reduce passive atrial emptying leading to a compensatory increase in active LA emptying. As people reach their 80 s, there is a plateau in the active emptying compensation^9,43^. This often occurs concurrently within interstitial atrial fibrosis. As LA compliance decreases, the compensatory increase in active LA emptying decreases. Atrial enlargement occurs as LA stroke volume increases^41^. Such changes have not been definitively proven in aging healthy dogs but could explain some of the ECG changes identified in this report.

In addition to the P wave changes, it was noted that R wave amplitude increased linearly with weight until about 18 pounds and then declined. It had a non-linear relationship with age and declined from 3.5 to 8 years of age before steadily increasing. This in agreement with a study evaluating ECG changes in military working dogs^19^. The R wave represents depolarization of the ventricular myocardium from the endocardial surface to the epicardial surface^44^. The exact of cause of this decrease in the younger age group is not known. Previously other studies have shown age-related changes in the R wave amplitude occur in neonatal puppies^45^. After birth there is an increase in the R wave amplitude as there is a predominance of the right ventricle after birth. Left ventricular development follows and begins to dominate after the first week of life due to an increase in left ventricular mass^31,46,47^. The clinical significance of the changes in R wave amplitude in this middle-aged cohort are not known.

Unique to veterinary medicine is the vastly different sizes of various breeds of dogs. Differences in heart rate and ECG waveforms have been previously evaluated in several different breeds. Generally, no significant differences in these variables between breeds have been observed although previous studies have alluded to higher R and Q wave amplitudes in larger breeds^20,21^. Hypothetically, this is due to the differences in ventricle size and thoracic conformations between different breeds. In this study, when evaluating for different breeds, we observed a weak association between mean weight of the breed category and mean age (Supplemental Figure S3), with smaller dogs (Chihuahua, Pomeranian, etc.) have a higher mean age than larger dogs (Great Dane, Bernese Mountain Dog, etc., Supplemental Figure S3). An exploratory analysis on the two most populous breeds showed similar associations between the five ECG waveforms and the covariates (Supplementary Figures S3–S11).

In this study a non-linear relationship was noted when evaluating QRS with age. Some studies in humans have shown a possible higher risk of sudden cardiac death with a prolonged QRS in addition to other ECG changes in both people with and without concurrent cardiac disease^48,49^. It is hypothesized that the prolonged QRS could cause abnormal depolarization and potentially facilitate reentrant tachyarrhythmias^48^. A prolonged QRS has also been identified in athletic or working dogs. In such studies, age was positively associated with a prolonged QRS duration^50,51^. Changes in QRS duration is part of a constellation of changes seen in endurance athletes and have been noted in horses and working dogs showing evidence of left ventricular enlargement^52,53^. Such studies have also shown a relationship between QRS duration and heart weight with longer durations seen in horses and greyhounds^52,53^. Cardiac hypertrophy results in longer pathways for electrical activation and repolarization of the ventricular myocardium. In human athletes, cardiac hypertrophy can be accompanied by changes in the autonomic system with a reduction in the sympathetic drive to the ventricles and a relative increase in vagal tone^52,53^. This study did not include evaluation of the athletic status of the dogs and as such the importance of this prolonged QRS still within the reference range of canines is not known.

Some sex related differences were also noted where intact male dogs had a shorter P wave duration than females. Few studies have shown significant differences between men and women with respect to P wave amplitude and duration although a slight trend of increased P wave duration in men than women and this duration increasing with advancing age has been found^54,55^. The opposite was found here where intact male dogs had a shorter P wave duration than females. One other veterinary study evaluating cardiac arrhythmias in a cohort of dogs showed that low voltage QRS was most commonly noted and also more commonly seen in female dogs versus males^56^. The effects of sex hormones on ventricular repolarization has been studied in humans and can parallel the rise of testosterone during puberty and subsequent decline in geriatric males^9,41,57–60^. Repolarization has been shown to be faster when comparing normal males to castrated males and in virilized women compared to normal women^41,53,54^. The canine population, secondary to common veterinary practices of spaying and neutering, can allow future studies to compare intact and neutered male and female cardiac changes.

Interestingly, both P and R wave amplitude had an association with increasing levels of anxiety. Several studies in humans evaluating ECG changes were performed during dental extractions and showed that anxiety commonly led to tachycardias^61^. One study in adult males showed an increased PWD with elevated levels of anxiety^55^. Excessive anxiety in humans may be associated with autonomic imbalances as increased sympathetic activity causes an increase in PWD^55^. With stress, activation of the sympathetic nervous system leads to elevations in catecholamines which can lead to atrial fibrosis^55,62^. Most dogs are naturally anxious at veterinary visits due to the new environment and odors, and changes in the heart rates in such situations are common^55^. Aside from administration of medications to specifically reduce anxiety and/or introduction of fear free veterinary practices, this is a difficult factor to control for; however, it is important that veterinarians know that anxiety can cause changes on the surface ECG. The effects of chronic anxiety and its effects on catecholamine levels would be difficult to evaluate in healthy dogs although the levels of these hormones have been found to increase with both preclinical and clinical dilated cardiomyopathy^55^.

Limitations to the study are inherent to the retrospective study design and the use of big data. There was a large subset of ECGs removed from analysis as the investigators were attempting to select primarily for healthy dogs by removing those with known cardiac conditions such as heart murmurs, gallops, or arrhythmias. In addition, positioning was restricted to the right lateral position as positioning has been shown to affect ECG amplitudes in dogs^63^. There was a large number of different weights of the dogs related to the multiple breeds included. This could affect the ability to draw direct parallels to humans where there can be less degree of different of weight within cohorts of adult people. Clinically significant myxomatous mitral valve disease is unlikely without an auscultable heart murmur although this is dependent on clinician’s auscultation ability and thus it is possible that a small population of dogs with myxomatous valve disease was inadvertently included into the analysis thereby affecting results. An additional limitation is the potential inclusion of dogs with occult dilated cardiomyopathy, a condition that cannot be ruled out based on the absence of a heart murmur. However, DCM is characterized by left ventricular dilatation, which is reported to cause an increase in R wave amplitude. The decreased in R wave amplitude identified in this study suggests occult DCM was not a major confounding limitation. Echocardiograms were not performed on the dogs evaluated, and the ECG can be an insensitive tool for detecting some anatomical cardiac changes. Anxiety level is also a subjective assessment and veterinarians are likely to grade degrees of anxiety differently depending on their individual opinions and clinical experience.

## Conclusions

This study showed that, similar to people, ECG changes associated with advancing age occur in dogs. While the changes were subtle and still within the reference range, the correlation was significant. Further studies evaluating echocardiographic parameters between young and old dogs to evaluate for potential explanations for these ECG findings appears warranted as they may be able to be used as a model for similar age-related changes in humans.

## Method

A cross sectional, retrospective design was used in the study. All sample results and animal clinical information were obtained from practicing veterinarians during their normal care of the animals and with the consent of the animals’ owners. IDEXX’s right to use the data for this study was granted under IDEXX’s customer agreements for reference laboratory services. Each study sample was obtained and submitted to an IDEXX Vetmedstat™ System by a practicing veterinarian during the routine diagnostic workup or monitoring system from May through September 2020. Each ECG result was identified by the breed, age, sex, anxiety level, heart rate and bodyweight of the dog from which the ECG was obtained. To ensure privacy, demographic information on the pet, pet owner, or veterinarian who submitted the sample was not collected. ECGs from a random sample of 50,000 individual dogs were eligible for inclusion into the study. Dogs were excluded for the following reasons: abnormal MEA, ECG not performed in right-lateral recumbency, heart murmur present, arrhythmia noted, sedatives (including alpha-2 agonists) given, atropine given, gender not reported, gallop present, age not reported, anti-arrhythmics given, weight not reported, P wave duration not reported, P wave amplitude not reported, QRS duration not reported, R amplitude not reported, heart rate not reported, anxiety level not reported, and not ausculted. A table of exclusion criteria can be found in supplementary Table S1 and an attribute plot for the largest intersections can be found in supplementary Figure S1.

## Supplementary Information

Supplementary Information.

## Data Availability

All data referenced in this study can be made available on request.
