# Supplementary material for: Assessment of age, gender, and anxiety on ECG waveform morphology in a large population of domestic dogs
Source: Sci Rep. 2022 May 5;12:7339. doi: 10.1038/s41598-022-11378-3 (PMC9072377; doi:10.1038/s41598-022-11378-3)
Supplement: Supplementary file 1 — Supplementary Information. [file 41598_2022_11378_MOESM1_ESM.pdf]

APPENDIX:

Supplementary Table 1. Attribute plot for exclusion criteria.

Exclusion Criteria	Percent	N
Abnormal MEA	31.7%	11,328
Not ausculted	21.3%	7,607
Not Lateral Recumbent	17.4%	6,228
Heart Murmur Present	8.7%	3,126
Arrhythmia Noted	4.9%	1,737
Sedatives Given	4.4%	1,569
P Duration Not Reported	3.2%	1,133
P Amplitude Not Reported	2.9%	1,024
Atropine Given	2.1%	746
Gender Not Reported	1.0%	354
Alpha 2 Given	1.0%	349
Gallop Present	0.5%	166

Age Not Reported	0.4%	128
Anti-Arrhythmics Given	0.3%	107
Weight Not Reported	0.2%	56
QRS Duration Not Reported	0.1%	27
R Amplitude Not Reported	0.1%	26
Heart Rate Not Reported	0.1%	23
Anxiety Level Not Reported	<0.1%	5

Supplemental Table 2. Descriptive statistics for the top eight dog breeds for age (years), heart rate (BPM), MEA (degree), weight (Lbs), Gender, and anxiety. Continuous variables reported with median and interquartile range (IQR). Categorical variables reported at percent and sample size.

	Mixed Breed (2,941)	Chihuahua (707)	Labrador Retriever (675)	Yorkshire Terrier (540)	Shih Tzu (497)	Dachshund (467)	Golden Retriever (457)	American Pit Bull Terrier (360)
Age (Year), median (IQR)	7.7 (2.2 - 10.5)	9.0 (6.8 - 11.4)	7.5 (2.2 - 10.1)	8.2 (5.0 - 10.8)	9.1 (6.1 - 11.6)	9.6 (7.0 - 12.1)	5.1 (1.0 - 9.4)	7.2 (4.2 - 9.8)
Heart Rate (BPM), median (IQR)	132.0 (113.0 - 150.0)	139.0 (122.0 - 160.0)	123.0 (107.0 - 140.0)	136.0 (117.0 - 158.2)	131.0 (115.0 - 149.0)	136.0 (117.0 - 159.0)	116.0 (101.0 - 131.0)	134.0 (121.0 - 154.0)
MEA (degree), median (IQR)	67.0 (55.0 - 78.0)	60.0 (50.0 - 72.0)	72.0 (60.0 - 83.0)	63.0 (53.0 - 74.0)	67.0 (57.0 - 78.0)	67.0 (53.0 - 77.0)	77.0 (66.0 - 84.0)	61.0 (52.0 - 73.0)
Weight (Lb), median (IQR)	34.8 (17.2 - 58.4)	9.4 (7.0 - 12.6)	73.0 (62.0 - 84.1)	8.0 (6.0 - 11.3)	14.0 (11.4 - 17.6)	14.9 (12.0 - 18.0)	70.5 (60.0 - 81.0)	63.1 (53.4 - 73.0)

**Gender, % (n)**								
Female	20.6% (607)	19.8% (140)	24.1% (163)	19.4% (105)	17.7% (88)	15.6% (73)	24.3% (111)	18.6% (67)
Female, Spayed	29.8% (875)	33.1% (234)	27.3% (184)	29.1% (157)	33.4% (166)	33.4% (156)	26.0% (119)	35.0% (126)
Male	21.6% (634)	15.8% (112)	25.2% (170)	20.9% (113)	17.9% (89)	15.0% (70)	31.7% (145)	19.4% (70)
Male, Neutered	28.1% (825)	31.3% (221)	23.4% (158)	30.6% (165)	31.0% (154)	36.0% (168)	17.9% (82)	26.9% (97)
**Anxiety, % (n)**								
Not anxious/very calm	6.4% (188)	5.5% (39)	10.2% (69)	5.9% (32)	7.0% (35)	8.8% (41)	18.2% (83)	6.4% (23)
Average Anxiety	61.6% (1812)	60.4% (427)	60.6% (409)	60.7% (328)	66.2% (329)	65.7% (307)	59.5% (272)	63.3% (228)

Very anxious or nervous	32.0% (941)	34.1% (241)	29.2% (197)	33.3% (180)	26.8% (133)	25.5% (119)	22.3% (102)	30.3% (109)

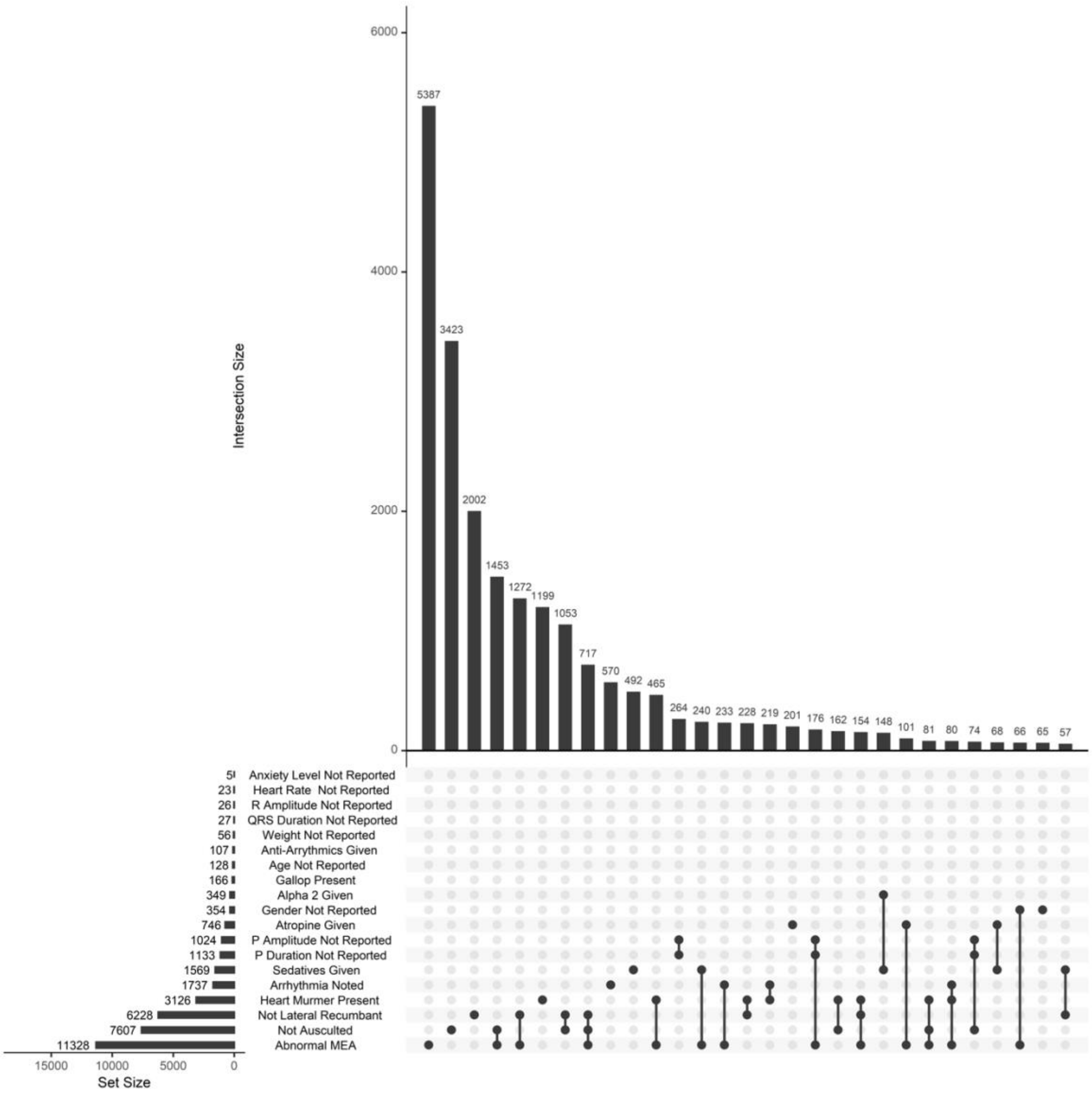
Intersection Size
6000
4000
2000
0
5387
3423
2002
1453
1272
1199
1053
717
570
492
465
264
240
233
228
219
201
176
162
154
148
101
81
80
74
68
66
65
57
5 Anxiety Level Not Reported
23 Heart Rate Not Reported
26 R Amplitude Not Reported
27 QRS Duration Not Reported
56 Weight Not Reported
107 Anti-Arrythmics Given
128 Age Not Reported
166 Gallop Present
349 Alpha 2 Given
354 Gender Not Reported
746 Atropine Given
1024 P Amplitude Not Reported
1133 P Duration Not Reported
1569 Sedatives Given
1737 Arrhythmia Noted
3126 Heart Murmer Present
6228 Not Lateral Recumbant
7607 Not Ausculted
11328 Abnormal MEA
15000
10000
5000
0
Set Size

Supplementary Figure S1. Attribute plot for exclusion criteria. Only intersections with a size greater than 50 (n > 50) are shown.

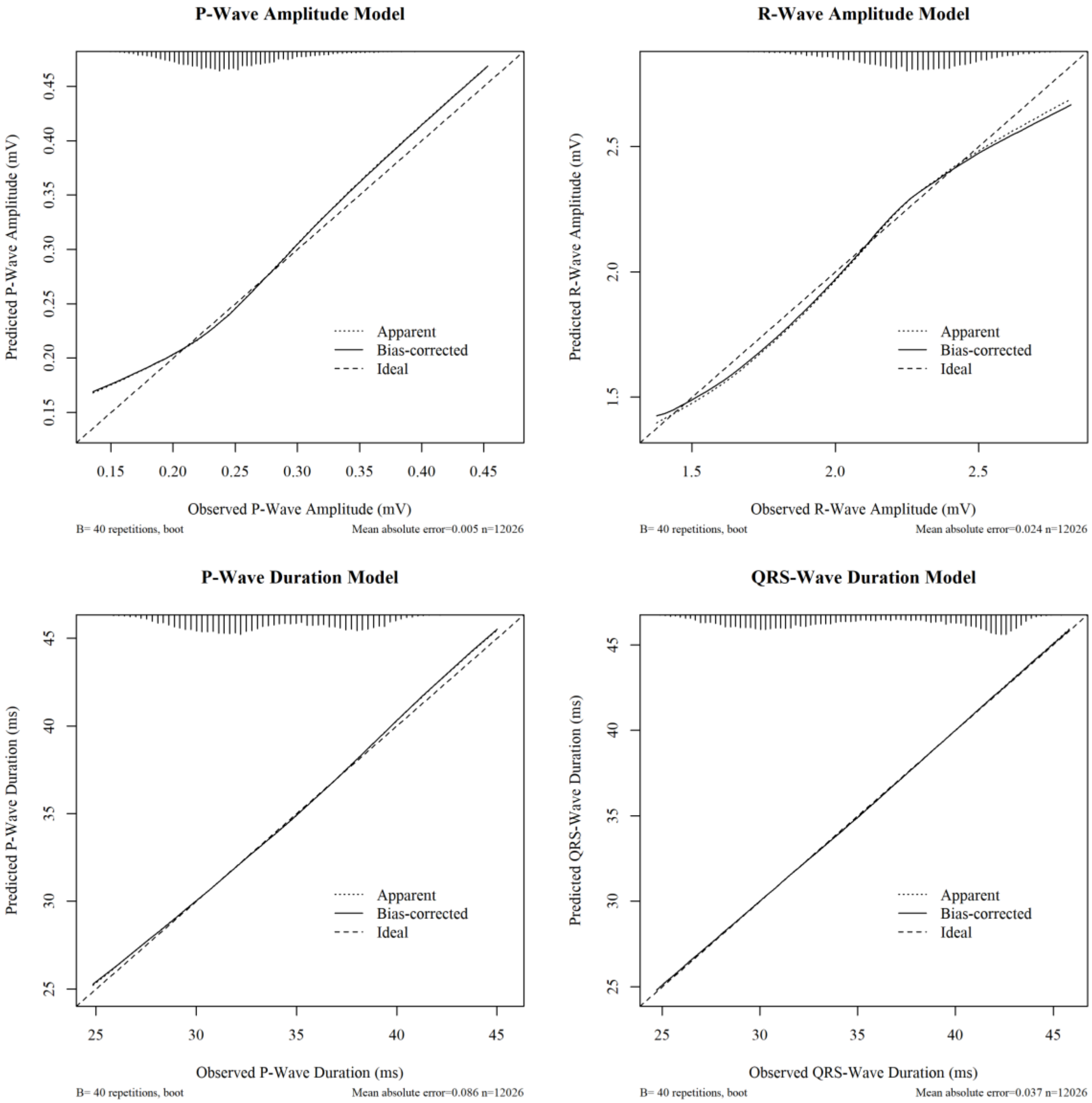

Supplementary Figure S2. Calibration curves of the four ECG parameter models (Top Left: P-Wave Amplitude, Top Right: R-Wave Amplitude, Bottom Left: P-Wave Duration, Bottom Right: QRS Duration). Calibration Curves for the four ECG parameter models. The dashed black line represents a perfect "ideal" fit (slope = 1.0, intercept = 0.0), the dotted line represents the actual fit and the solid line represents the bias-corrected fit. At the top of the figure is a histogram of the observed ECG waveform measurements.

Supplemental Methods

At the request of the reviewers, a *post-hoc* exploratory analysis of the five ECG waveform models was conducted with the addition of breed as a covariate. Multivariable-adjusted linear regression analyses were used to model a flexible association between each of the five ECG waveform measurements (P amplitude, R amplitude, P-wave duration, R-wave duration, and QRS-wave duration). Continuous covariates consisted of age, weight, MEA, and heart rate (all modeled using restricted cubic splines). Categorical covariates consisted of reported anxiety level (three levels), sex (two levels), and breed (183 levels). Knot selection for restricted cubic splines was previously described (see methods). Compared to the pre-defined models described in the study, the addition of the breed variable led to an increase in AIC (i.e. the increase in model complexity decreased model

fit and performance). Only model output for the two most populous breeds: Chihuahua (Supplemental Figures S4-S7) and Labrador Retrievers (Supplemental Figures S8-S11) are shown. Due to the differences in size of breeds shown, only the range of the data for weight and age were used when plotting the model output. For Chihuahuas the range of age was 0.5 to 14.6 years, and the range of weight was 0.8 to 15.8 lbs. For Labrador Retrievers the range of age was 1.0 to 13.8 years, and the range of weight was 4.8 to 106 lbs.

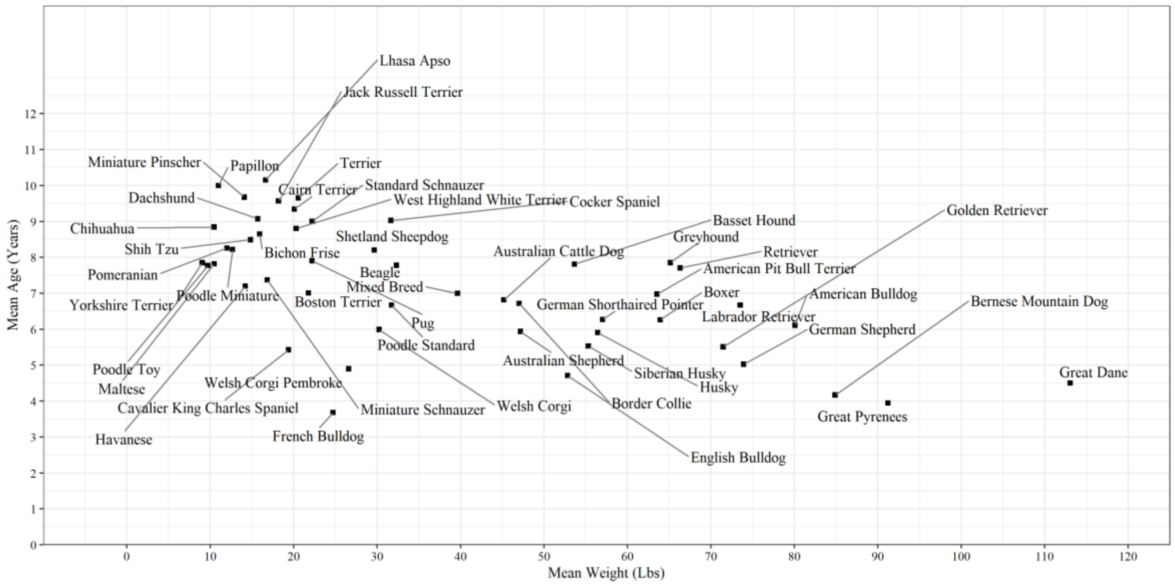

Supplemental Figure S3. Scatter plot of the mean age (years) and mean weight (Lbs) by dog breed. Only breed categories with a sample size > 40 records were plotted to ensure visibility.

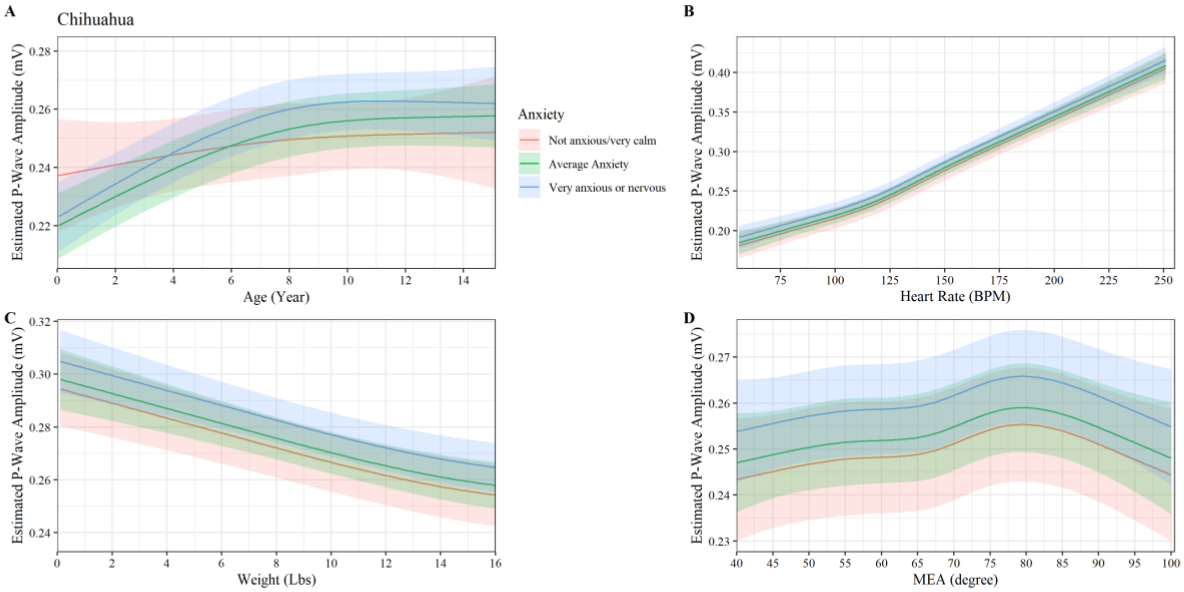

Supplemental Figure S4. Plot of the p-wave amplitude (mV) versus A) age, B) heart rate, C) weight, and D) MEA by anxiety for "Chihuahua" breed category. The y axis shows the estimated p-wave amplitude from the fully adjusted model for observed values while holding the values of covariates at their referent values. The curves display the trend in p-wave amplitude with increase age, heart rate, weight, and MEA. Shaded regions represent the 95% confidence interval of the prediction estimates from the regression model.

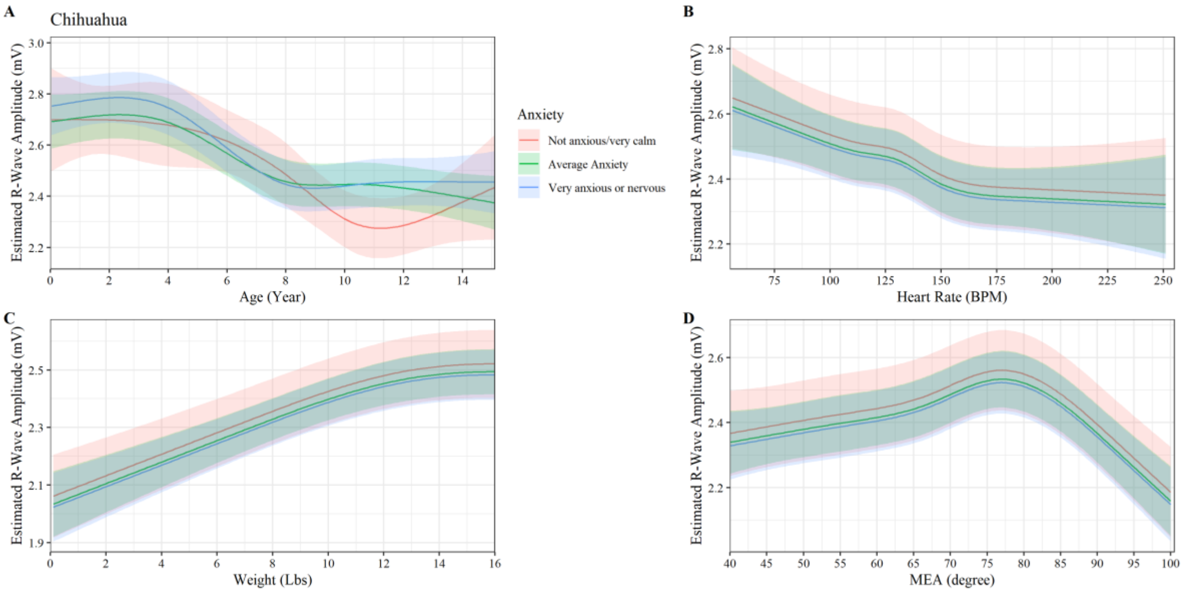

Supplemental Figure S5. Plot of the r-wave amplitude (mV) versus A) age, B) heart rate, C) weight, and D) MEA by anxiety for "Chihuahua" breed category. The y axis shows the estimated r-wave amplitude from the fully adjusted model for observed values while holding the values of covariates at their referent values. The curves display the trend in r-wave amplitude with increase age, heart rate, weight, and MEA. Shaded regions represent the 95% confidence interval of the prediction estimates from the regression model.

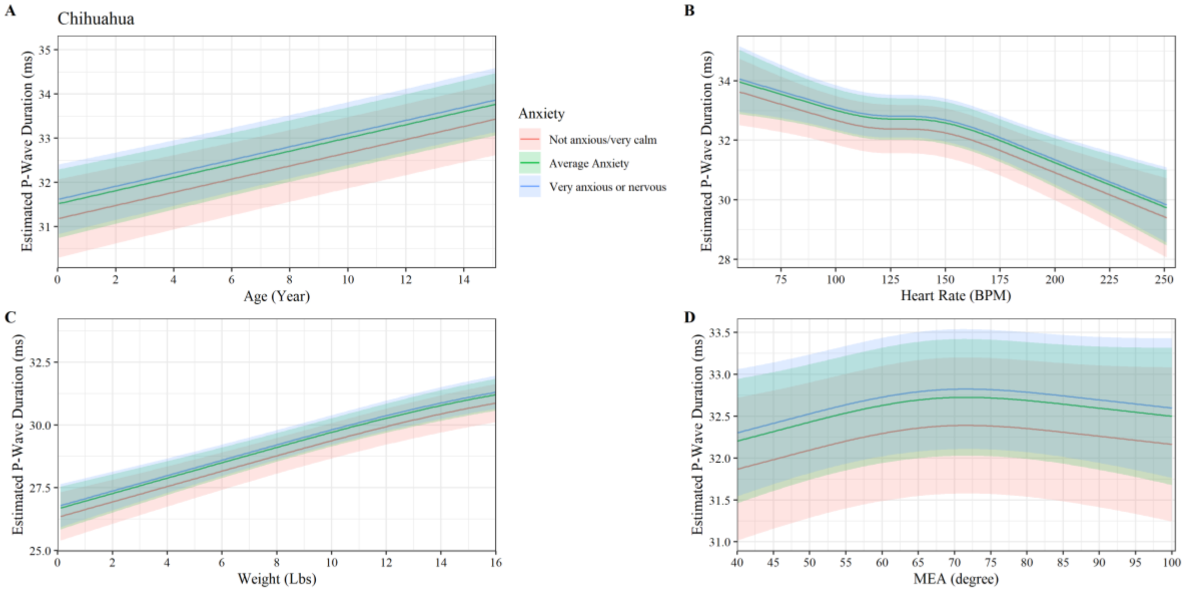

Supplemental Figure S6. Plot of the p-wave duration (ms) versus A) age, B) heart rate, C) weight, and D) MEA by anxiety for "Chihuahua" breed category. The y axis shows the estimated p-wave duration from the fully adjusted model for observed values while holding the values of covariates at their referent values. The curves display the trend in p-wave duration with increase age, heart rate, weight, and MEA. Shaded regions represent the 95% confidence interval of the prediction estimates from the regression model.

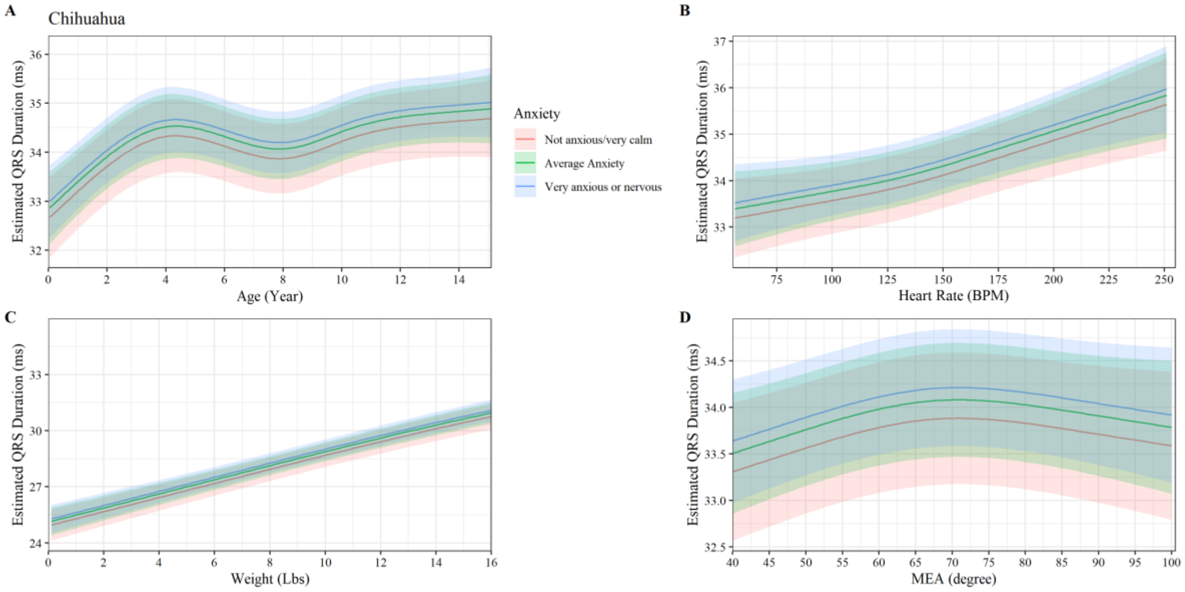

Supplemental Figure S7. Plot of the QRS-wave duration (ms) versus A) age, B) heart rate, C) weight, D) MEA by anxiety for "Chihuahua" breed category. The y axis shows the estimated QRS - wave duration from the fully adjusted model for observed values while holding the values of covariates at their referent values. The curves display the trend in QRS-wave duration with increase age, heart rate, weight, and MEA. Shaded regions represent the 95% confidence interval of the prediction estimates from the regression model.

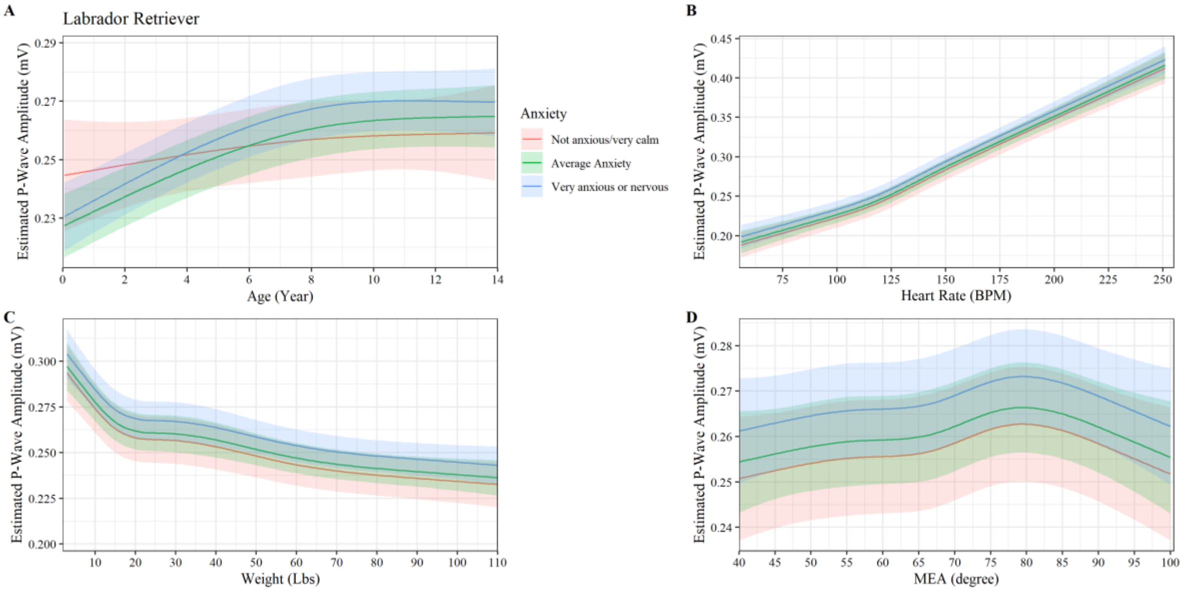

Supplemental Figure S8. Plot of the p-wave amplitude (mV) versus A) age, B) heart rate, C) weight, and D) MEA by anxiety for "Labrador Retriever" breed category. The y axis shows the estimated p-wave amplitude from the fully adjusted model for observed values while holding the values of covariates at their referent values. The curves display the trend in p-wave amplitude with increase age, heart rate, weight, and MEA. Shaded regions represent the 95% confidence interval of the prediction estimates from the regression model.

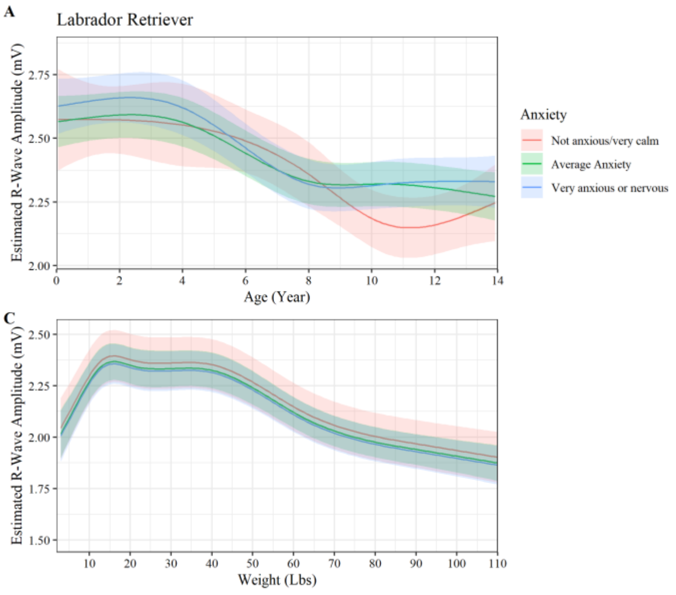

Supplemental Figure S9. Plot of the r-wave amplitude (mV) versus A) age, B) heart rate, C) weight, and D) MEA by anxiety for "Labrador Retriever" breed category. The y axis shows the estimated r-wave amplitude from the fully adjusted model for observed values while holding the values of covariates at their referent values. The curves display the trend in r-wave amplitude with increase age, heart rate, weight, and MEA. Shaded regions represent the 95% confidence interval of the prediction estimates from the regression model.

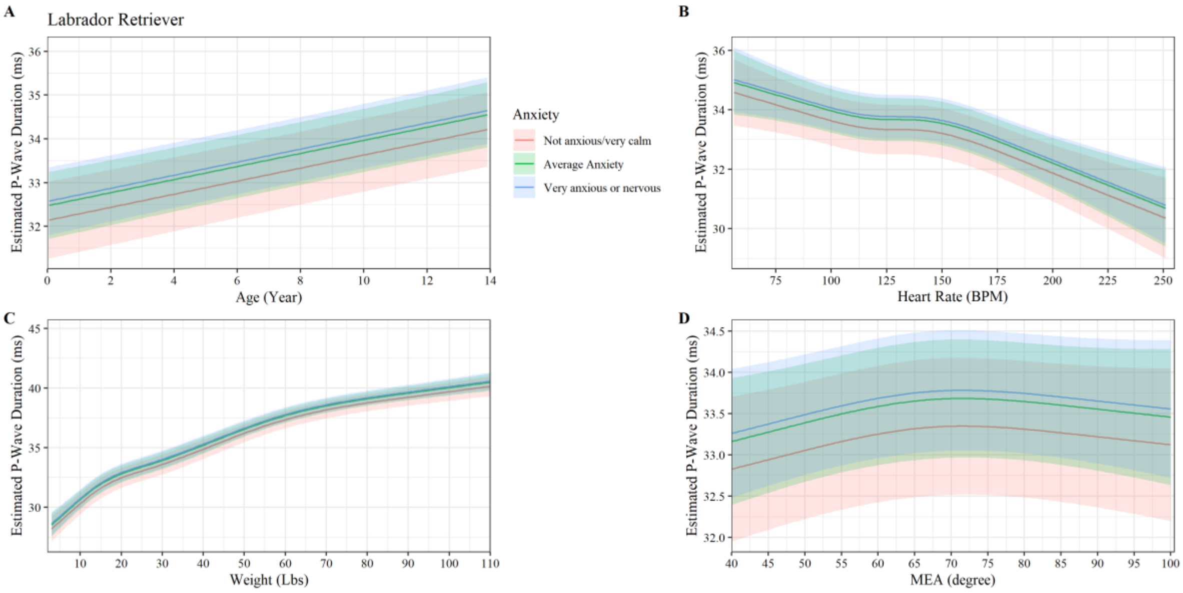

Supplemental Figure S10. Plot of the p-wave duration (ms) versus A) age, B) heart rate, C) weight, and D) MEA by anxiety for "Labrador Retriever" breed category. The y axis shows the estimated p-wave duration from the fully adjusted model for observed values while holding the values of covariates at their referent values. The curves display the trend in p-wave duration with increase age, heart rate, weight, and MEA. Shaded regions represent the 95% confidence interval of the prediction estimates from the regression model.

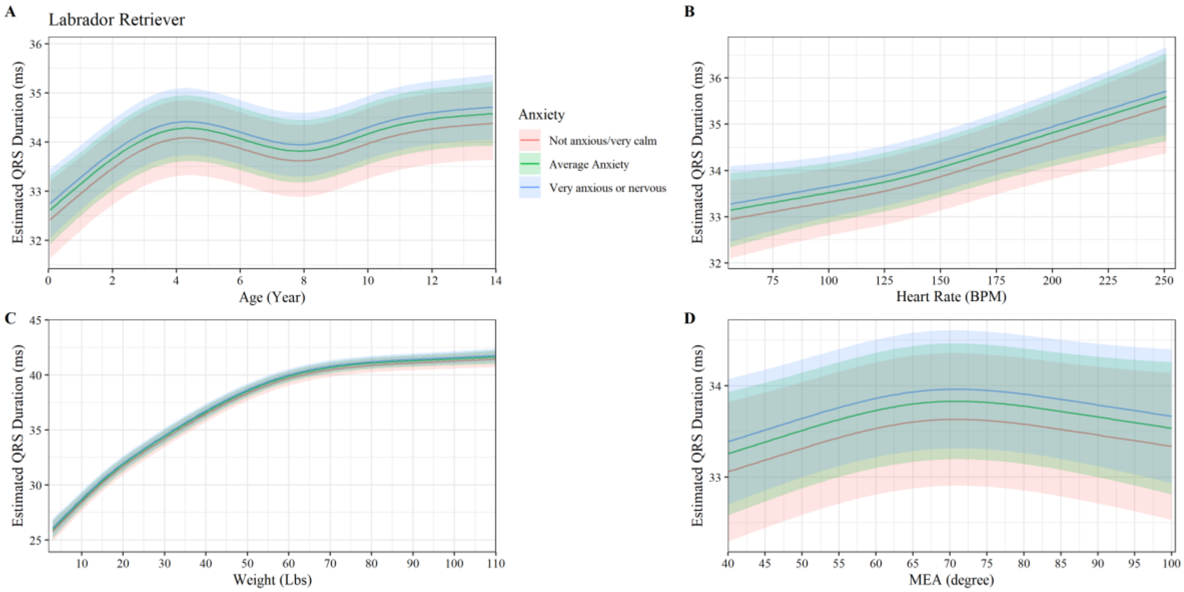

Supplemental Figure S11. Plot of the QRS-wave duration (ms) versus A) age, B) heart rate, C) weight, D) MEA by anxiety for "Labrador Retriever" breed category. The y axis shows the estimated QRS -wave duration from the fully adjusted model for observed values while holding the values of covariates at their referent values. The curves display the trend in QRS-wave duration with increase age, heart rate, weight, and MEA. Shaded regions represent the 95% confidence interval of the prediction estimates from the regression model.
